# Advances in surgical treatment of chronic pancreatitis

**DOI:** 10.1186/s12957-014-0430-4

**Published:** 2015-02-08

**Authors:** Qingqiang Ni, Lin Yun, Manish Roy, Dong Shang

**Affiliations:** Medical College of Soochow University, No.199 Renai Road, Suzhou, Jiangsu 215123 People’s Republic of China; Eastern Hepatobiliary Surgery Hospital, No.225 Changhai Road, Shanghai, 200433 People’s Republic of China; Department of General Surgery, Pancreato-Biliary Center, First Affiliated Hospital, Dalian Medical University, No.222 Zhongshan Road, Dalian, Liaoning 116011 People’s Republic of China; Jinan Maternity and Child Care Hospital, No.2 Jianguoxiaojing 3 Road, Jinan, Shandong 250001 People’s Republic of China; Department of Surgery, Patan Academy of Health Science Lagankhel, PO Box 26500, Lalitpur, 44700 Nepal

**Keywords:** Chronic pancreatitis, Surgery, Treatment

## Abstract

The incidence of chronic pancreatitis (CP) is between 2 and 200 per 100,000 persons and shows an increasing trend year by year. India has the highest incidence of CP in the world at approximately 114 to 200 per 100,000 persons. The incidence of CP in China is approximately 13 per 100,000 persons. The aim of this review is to assist surgeons in managing patients with CP in surgical treatment. We conducted a PubMed search for “chronic pancreatitis” and “surgical treatment” and reviewed relevant articles. On the basis of our review of the literature, we found that CP cannot be completely cured. The purpose of surgical therapy for CP is to relieve symptoms, especially pain; to improve the patient’s quality of life; and to treat complications. Decompression (drainage), resection, neuroablation and decompression combined with resection are commonly used methods for the surgical treatment of CP. Before developing a surgical regimen, surgeons should comprehensively evaluate the patient’s clinical manifestations, auxiliary examination results and medical history to develop an individualized surgical treatment regimen.

**Electronic supplementary material:**

The online version of this article (doi:10.1186/s12957-014-0430-4) contains supplementary material, which is available to authorized users.

## Introduction

*Chronic pancreatitis* (CP) refers to limiting, segmental, diffusing, progressive inflammatory damage, necrosis, and interstitial fibrous lesions of pancreatic parenchyma caused by many different factors, usually accompanied by stenosis and dilation of the pancreatic duct, pancreatic calcification, and pancreatic stone formation. The necrosis of pancreatic acinar cells, the atrophy or loss of pancreatic islet cells, and extensive interstitial fibrosis will eventually result in the irreversible destruction of pancreatic morphology and structure as well as exocrine and endocrine pancreatic insufficiency.

Globally, the incidence of CP is between 2 and 200 per 100,000 persons and shows an increasing trend year by year [1,2]. India has the highest incidence of CP in the world at approximately 114 to 200 per 100,000 persons [2]. The incidence of CP in China is approximately 13 per 100,000 persons [1,2]. It has been reported that approximately 90% of patients with CP have symptoms of abdominal pain [3,4]. Although the mechanism of abdominal pain is still not clear, pancreatic duct hypertension, pancreatic inflammation, and peripancreatic immune cell infiltration usually play important roles in the mechanism of abdominal pain in CP [4,5].

Historically, excess alcohol consumption plays a leading role in Western countries, accounting for 60% of CP cases. In China, excess alcohol consumption accounts for approximately 35% of the cases of CP [6]. According to a 2007 multicenter survey on CP in China, CP caused by biliary tract diseases accounted for 33.9% of cases; CP caused by long-term excess alcohol consumption accounted for 35.4%; and CP caused by pancreatic trauma accounted for 10.5% [7]. In China, with the continuous improvement of quality of life, excess alcohol consumption has gradually replaced biliary tract diseases to become the leading cause of CP.

On the basis of the histopathological changes in the pancreas, CP can be classified into three types: (1) chronic obstructive pancreatitis; (2) chronic calcifying pancreatitis, the most common type of CP, which includes alcoholic CP; and (3) chronic inflammatory pancreatitis, including CP resulting from chronic inflammation of the biliary tract and stenosis induced by scar formation [8].

Several studies have shown that for the treatment of patients with CP, surgical treatment is better than endoscopic treatment [9-14]. Surgical intervention is usually required when the history of CP is several years or even decades; the treatment effect of internal medicine and endoscopy is poor; abdominal pain is intractable; pancreatic cancer cannot be excluded before surgery; or there are complications involving adjacent organs, such as duodenal stenosis, bile duct stricture, portal vein stenosis combined with portal vein hypertension, pancreatic necrosis, pancreatic pseudocyst, or pancreatic fistula [15,16]. Pancreatic necrosis can be classified as pancreatic parenchymal necrosis alone, peripancreatic tissue necrosis, or both.

CP cannot be completely cured. The purpose of surgical therapy for CP is to relieve symptoms, especially pain; to improve the patient’s quality of life; and to treat complications [17]. Generally speaking, decompression (drainage), resection, neuroablation, and decompression combined with resection are commonly used methods for the surgical treatment of CP [18]. The methods of decompression and surgical resection are reviewed below.

## Review

### Decompression

When the main pancreatic duct is dilated more than 7 mm and there is no inflammatory pseudotumor at the pancreatic head, then pancreatic duct hypertension or parenchymal hypertension may be the mechanism causing pain. Performing decompression can achieve satisfactory pain symptom relief [19,20].

### DuVal procedure

In 1954, to resolve the pancreatic hypertension problem in patients with CP, DuVal [21] and Zollinger et al. [22] independently conducted main pancreatic duct decompression. They performed pancreatic tail resection and end-to-end or end-to-side pancreaticojejunostomy of the residual pancreatic end and jejunum to achieve retrograde drainage of the pancreatic duct. However, this method is applicable only to the obstruction of the pancreatic duct at the pancreatic head and dilation of the whole main pancreatic duct. This method is prone to cause postoperative pancreatic duct stump stenosis; its effect on stenosis at other pancreatic duct locations is not good; and the recurrence of pain symptoms is common. Therefore, this method is rarely used in clinical practice.

### Puestow-Gillesby procedure

In 1958, to solve the problem of recurrent multiple strictures of the pancreatic duct in the DuVal procedure, Puestow and Gillesby first proposed the Puestow-Gillesby procedure [23]. In this procedure, the spleen and pancreatic tail are resected, and the pancreatic duct is longitudinally opened. The longitudinally opened pancreatic duct is then connected with the jejunum via side-to-side pancreaticojejunostomy to drain the pancreatic juice and reduce the incidence of late-stage strictures. The Puestow-Gillesby procedure is used primarily in cases of multiple strictures or stones in the pancreatic duct. However, this complex procedure usually results in severe trauma to patients with CP; therefore, it is not extensively applied in clinical practice.

### Partington-Rochelle procedure

Two years after Puestow and Gillesby proposed the Puestow-Gillesby procedure, Partington and Rochelle modified the Puestow-Gillesby procedure. In the Partington-Rochelle procedure, the whole pancreatic duct is longitudinally opened, and the whole opened pancreatic duct is connected with the jejunum via side-to-side pancreaticojejunostomy. The pancreatic tail and spleen are preserved [24]. The Partington-Rochelle procedure maximizes the preservation of the pancreatic tissues and minimizes the impact on the endocrine and exocrine functions of pancreas; thus, it is the most extensively applied CP decompression procedure in clinical practice. A randomized controlled trial showed that compared with endoscopic drainage, pancreaticojejunostomy (the Partington-Rochelle procedure) has the advantage of providing improvement of quality of life and pain relief [9]. It was reported that the postoperative mortality rate and disease incidence of patients with CP who underwent the Partington-Rochelle procedure were lower than among those who had the Puestow-Gillesby procedure, at approximately 3% and 20%, respectively [25]. Short-term follow-up showed that the pain relief rate after this decompression procedure was approximately 75%. However, its long-term pain relief benefit is still not ideal [25].

### Resection

#### Pancreatoduodenectomy

In 1935, Whipple *et al*. successfully performed pancreatoduodenectomy (PD) in one patient with an ampullary tumor [26]. In 1946, Whipple described the experience of applying proximal PD in the treatment of patients with chronic calcifying pancreatitis [27]. The resection range in this procedure includes the duodenum and one-third of the distal end of the stomach. This procedure has gradually become a classical proximal PD and is called the Whipple procedure. In 1935, this PD procedure was first used in a patient with a malignant tumor of the pancreatic head. Since the classical Whipple procedure was proposed in 1946 and with the continuous improvement in its safety, it has increasingly been applied in the treatment of patients with CP [28]. After more than a half century of application, the Whipple procedure has been confirmed as an effective method for treating pain and complications in CP. In the late 1970s, owing to the continuous improvement of perioperative preparations, the Whipple procedure was applied extensively. The results of three large-scale clinical trials showed that within 4 to 6 years after patients were surgically treated with the Whipple procedure, the pain relief rate reached 71% to 89%. Although the postoperative mortality rate was lower than 5%, the incidence of CP was maintained at approximately 40% [18,29-31].

#### Pylorus-preserving pancreatoduodenectomy

In 1978, Traverso and Longmire described their experience with performing pylorus-preserving pancreatoduodenectomy (PPPD) in patients with CP [32]. They considered the normal physiological function of gastric emptying, avoided complications caused by subtotal gastrectomy, and avoided the loss of gastric digestion and absorption function, thus improving the problem of postoperative malnutrition [32-34]. The resection range of the PPPD procedure is essentially the same as that of the classical Whipple procedure. During surgery, the duodenum is transected at 2 cm from the pylorus. The right gastric artery, main gastroduodenal artery and corresponding vagus nerve, and right gastroepiploic artery are preserved. In addition, the stomach, pylorus, and duodenal bulb 1.5 to 2 cm below the pylorus are also preserved. The long-term pain symptoms of 90% of patients can be improved after the PPPD procedure [35,36]. It was reported that temporary gastric emptying disorders are among the major postoperative complications [36]. For experienced surgeons, the Whipple procedure and the PPPD procedure are safe and effective, and the surgical mortality rate is only 2% to 5%, with a high long-term pain relief rate [19,37].

#### Distal pancreatectomy

When patients with CP have left pancreatic duct rupture or suspected malignant tumor masses and the diameter of the main pancreatic duct is smaller than 5 mm, distal pancreatectomy (DP) is usually performed. In 1882, Trendelenburg performed the first DP in one patient with a pancreatic tumor [38]. In 1913, the Mayo Clinic formulated the standard DP procedure [39]. The conventional resection range of the distal pancreas includes the pancreatic body and pancreatic tail. One of the most common complications after DP is pancreatic stump fistula. Approximately 0% to 7% of patients have this complication after undergoing DP [40-43]. The incidence of diabetes mellitus in patients with CP after DP is 17% to 85% [44-47]. This procedure has a large resection range on the pancreas, and the surgery can affect endocrine and exocrine functions of the pancreas to different degrees; therefore, it should be carefully considered.

#### Middle segment pancreatectomy

When the lesion is at the pancreatic neck and proximal pancreas, middle segment pancreatectomy (MSP) is the third surgical method, in addition to the Whipple procedure and DP procedure. MSP was first proposed by Guillemin and Bessot in 1957 in a report on their use of MSP combined with pancreaticojejunostomy in a patient with CP [48].

The advantages of MSP are the preservation of most normal pancreatic tissues, low incidence of postoperative endocrine and exocrine insufficiency, maintenance of digestive tract continuity, and preservation of the spleen [49,50]. Warshaw *et al*. reported performing MSP in patients with CP and have not yet observed diabetes mellitus as a complication, and no reports of diabetes mellitus complications were found from Warshaw after 1998 [51]. Roggin *et al*. analyzed 207 patients and showed that MSP had a postoperative recurrence rate of 33% and an incidence of pancreatic fistula of 22.2%. The incidence of pancreatic fistula in MSP was higher than in PD and DP [52]. The indications for MSP application are narrow; the manipulation involved in the procedure is complex; and experienced pancreatic surgeons are required. Therefore, MSP needs to be chosen carefully.

#### Total pancreatectomy

In 1944, for the first time, Priestley *et al*. successfully treated a patient with hyperinsulinemia using total pancreatectomy (TP) [53]. As TP is associated with high rates of complications, mortality, and loss of pancreatic function, surgeons have largely abandoned it. TP is chosen only when CP involves the whole pancreas; there is insulin-dependent diabetes mellitus, pancreatic function is lost; there are pancreatic surgical complications (such as pancreatic fistula or anastomotic leakage); and there is intractable postoperative pain [54,55]. TP tends to result in brittle diabetes, for which treatment is difficult; hence, TP combined with islet autotransplantation (IAT) is often used to minimize or prevent TP-related diabetes. TP combined with IAT can improve the patient’s quality of life and reduce hypoglycemic episodes.

### Decompression combined with resection

#### Beger procedure

In the early 1970s, Beger, a German surgeon, observed that inflammatory masses were usually present at pancreatic head locations in many patients with CP. In 1972, Beger performed the first duodenum-preserving pancreatic head resection (DPPHR), also called the *Beger procedure*, in a patient with CP. Surgical experiences with the DPPHR procedure were summarized and published in 1980 after Beger had performed a total of 52 of them [56]. The key steps of DPPHR include transection of the pancreatic neck above the portal vein, resection of pancreatic head masses, preservation of the posterior branch of the gastroduodenal artery to retain the blood supply of the duodenum, and preservation of the integrity of the lower end of the common bile duct to obtain a decompression effect in the common bile duct and duodenum. The proximal pancreatic duct is ligated, and the distal end is used for pancreaticojejunostomy. If there is lower bile duct obstruction, choledochojejunostomy can also be performed. The advantage of DPPHR is the preservation of the physiological function of the stomach, duodenum, and common bile duct [57]. In some experienced hospitals, the mortality rate of this surgery is low (0% to 3%), and the recurrence rate is 15% to 32% [57,58]. The DPPHR procedure can achieve 75% to 95% long-term pain relief [58-60].

#### Frey procedure

In 1987, a new, modified type of DPPHR, local resection of the pancreatic head combined with a longitudinal pancreaticojejunostomy, was first reported by Frey [61]. Thus, it is also called the *Frey procedure*. The Frey procedure is a combination of the Beger procedure and Partington-Rochelle procedure. Compared with the Beger procedure, the Frey procedure has a smaller resection range of the pancreatic head. In addition, combined with side-to-side pancreaticojejunostomy, pancreatic juice can be drained along the pancreatic duct in the direction of the pancreatic tail. When patients with CP have pancreatic duct obstruction at the pancreatic head and pancreatic tail as well as small inflammatory masses at the pancreatic head, the Frey procedure can be considered. The Frey procedure is not applicable when there are large inflammatory masses at the pancreatic head and no stenosis of the left pancreatic duct [62]. A retrospective randomized controlled trial study showed that the recurrence rate after the Frey procedure (19%) was lower than that after PPPD (53%). Another study showed that the recurrence rate after the Frey procedure (22%) was lower than that after the Beger procedure (32%). The 7-year endocrine insufficiency rate after the Frey procedure (86%) was lower than that after PPPD (96%). The 8-year endocrine insufficiency rate after the Frey procedure (78%) was lower than that after the Beger procedure (88%) [57,58,63,64].

#### Berne modification

In 2001, to target the condition of portal hypertension in certain patients with CP, Gloor *et al*. modified the Beger procedure and the Frey procedure to develop the Berne procedure [65]. Resection of pancreatic tissues at the portal vein level is usually difficult because of inflammation or portal hypertension. In the Berne procedure, this resection is not performed. The Berne procedure has a pancreatic head resection range similar to the Beger procedure. The pancreatic neck is preserved, and Roux-en-Y anastomosis is performed between the pancreatic head and the jejunum. Farkas *et al*. reported a 10-month follow-up study on 30 patients who underwent the Berne procedure and found no severe complications [66]. In addition, pancreatic exocrine and endocrine functions were enhanced after the procedure. A study on 100 patients with CP who were surgically treated with the Berne procedure showed a low postoperative mortality rate (1%) and a low postoperative complication rate (16%). In the patient sample, 55% had improved postoperative pain, and 67% had increased body weight. The treatment effect of the Berne procedure on CP is very ideal [67]. The operative time and the length of hospitalization in patients who underwent surgery with the Berne procedure were both shorter compared with the PPPD and Beger procedures [68,69]. The Berne procedure is a mature, effective, and safe surgical procedure with a mortality rate of 0% to 1% and a recurrence rate of 20% to 23% [65].

#### Imaizumi modification

In 2009, Hatori *et al*. first proposed a modified Beger procedure called the *Imaizumi procedure* [70]. The Imaizumi procedure is a combination of the Beger procedure and DPPHR. When treating patients with CP with common bile duct stenosis, the Imaizumi procedure is particularly useful. Compared with the conventional Beger procedure, the Imaizumi procedure has a larger range of resection in the treatment of common bile duct stenosis in patients with CP that includes the intrapancreatic bile duct. More than 90% of patients experience postoperative pain relief. In addition, compared to the PPPD procedure, the rate of exocrine and endocrine pancreatic insufficiency associated with the Imaizumi procedure is lower, but the postoperative complications and mortality rates between these two exhibited no significant difference. When patients with CP have pancreatic head masses and bile duct stricture at the pancreatic head location, the Imaizumi procedure is a useful surgical treatment method.

#### Hamburg modification

In 1998, Izbicki *et al*. modified the Frey procedure and developed the Hamburg procedure [71]. This procedure is applicable to patients with CP who have thin pancreatic ducts smaller than 3 mm in diameter. This procedure has a large range of pancreatic head resection, and the central part of the uncinate process is also resected. The pancreatic tissues are resected in a V shape. This procedure is safe and effective and can significantly improve the patient’s postoperative quality of life and establish pain relief [72,73].

### Neuroablative procedure

The pancreatic sympathetic nerve, pancreatic head plexus, and pancreatic tail plexus jointly innervate the pancreas. Neuroablation can be considered for patients with CP without pancreatic duct dilation and pancreatic duct stones. When the lesion of a patient with CP is at the pancreatic head, a pancreatic head plexus resection can be performed. When the lesion of a patient with CP is at the pancreatic tail, splanchnicectomy and celiac ganglionectomy can be selectively performed. The neuroablative procedure has fewer complications, and the main presentations are gastric emptying disorder, enteroparalysis, and intestinal motility dysfunction. However, its effect on long-term pain relief is not ideal; therefore, the neuroablative procedure is rarely applied in clinical practice.

## Conclusions

During the surgical treatment of patients with CP, no matter what type of surgery is performed, the long-term postoperative pain relief rate, postoperative recurrence rate and mortality rate, postoperative quality of life, and postoperative changes in pancreatic endocrine and exocrine functions are all issues for clinical surgeons to consider. Therefore, before developing a surgical regimen, surgeons should comprehensively evaluate the patient’s clinical manifestations, auxiliary examination results, and medical history to develop an individualized surgical treatment regimen.

## Abbreviations

CP: Chronic pancreatitis
DP: Distal pancreatectomy
DPPHR: Duodenum-preserving pancreatic head resection
IAT: Islet autotransplantation
MSP: Middle segment pancreatectomy
PD: Pancreatoduodenectomy
PPPD: Pylorus-preserving pancreatoduodenectomy
TP: Total pancreatectomy
